# Galectin-3 Reflects Systemic Atherosclerosis in Patients with Coronary Artery Disease

**DOI:** 10.3390/medicina61081388

**Published:** 2025-07-30

**Authors:** Horea-Laurentiu Onea, Calin Homorodean, Florin-Leontin Lazar, Mihai Octavian Negrea, Teodora Calin, Ioan Cornel Bitea, Minodora Teodoru, Vlad Ionut Nechita, Ariela Ligia Olteanu, Dan-Mircea Olinic

**Affiliations:** 1 4th Department of Internal Medicine Medical Clinic No.1, Iuliu Hatieganu University of Medicine and Pharmacy, 400006 Cluj-Napoca, Romania; onea.lau@gmail.com (H.-L.O.); danolinic@gmail.com (D.-M.O.); 2 Department of Cardiology, County Clinical Emergency Hospital Sibiu, 550245 Sibiu, Romania; mihaioctavian.negrea@ulbsibiu.ro (M.O.N.); cornelioanbitea@yahoo.com (I.C.B.); minodora.teodoru@ulbsibiu.ro (M.T.); 3 2nd Department of Cardiology, County Clinical Emergency Hospital Cluj-Napoca, 400006 Cluj-Napoca, Romania; 4 Department of Clinical Medicine, Faculty of Medicine, “Lucian Blaga” University, 550024 Sibiu, Romania; 5 Department of Preclinical Medicine, Microbiology, Faculty of Medicine, “Lucian Blaga” University, 550024 Sibiu, Romania; teodora.calin65@gmail.com; 6 Department of Medical Informatics and Biostatistics, “Iuliu Hațieganu” University of Medicine and Pharmacy, 400349 Cluj-Napoca, Romania; nechita.vlad@umfcluj.ro; 7 Clinical Laboratory, County Clinical Emergency Hospital Sibiu, 550245 Sibiu, Romania; ariela.olteanu@scjus.ro

**Keywords:** coronary artery disease, acute coronary syndromes, Galectin-3, systemic atherosclerosis, carotid plaque, femoral plaque

## Abstract

*Background and Objectives*: Galectin-3 (Gal-3), a pro-inflammatory cytokine, has been implicated in atherosclerosis and adverse cardiovascular outcomes. While its role in coronary artery disease (CAD) is increasingly recognized, its association with systemic atherosclerosis remains underexplored. Objective: To investigate serum Gal-3 levels in patients with CAD and evaluate correlations between CAD severity and extra-coronary atherosclerotic involvement (carotid, femoral, and radial territories). *Materials and Methods*: We prospectively enrolled 56 patients with CAD undergoing coronary angiography (42.8% with acute-ACS; 57.2% with chronic coronary syndromes-CCS). Gal-3 levels were measured within 24 h of admission. Atherosclerosis severity was assessed angiographically and through vascular ultrasound of the carotid, femoral, and radial arteries. Patients were stratified by median Gal-3 levels, and clinical follow-up was performed at 1 and 3 months. *Results:* Gal-3 levels were significantly higher in CAD vs. controls (20.7 vs. 10.1 ng/mL; *p* < 0.00001) and in ACS vs. CCS (22.18. vs. 17.93 ng/mL; *p* = 0.019). Gal-3 correlated positively with culprit lesion diameter stenosis (DS) (R = 0.30; *p* = 0.023) and maximum severity of additional treated lesions (R = 0.62; *p* = 0.006). Gal-3 also correlated positively with carotid plaque thickness (R = 0.32; *p* = 0.016), while patients with Gal-3 levels above the median showed increased median values for femoral plaque thickness (32.4 vs. 26.45 mm, *p* = 0.046). No correlation was found with radial artery calcification. Gal-3 showed moderate discrimination for ACS (AUC = 0.685; cut-off 20.18 ng/mL). On multivariate analysis age, DS, and ACS presentation were independent predictors of Gal-3 above 19.07 ng/mL. *Conclusions:* Gal-3 levels are elevated in ACS and correlate with atherosclerotic burden, particularly in coronary, carotid, and femoral territories. These findings support Gal-3 as a potential marker of lesion severity and systemic vascular involvement, highlighting its possible role in risk stratification and the monitoring of atherosclerotic disease progression. This study provides integrated insights into the impact of Gal-3 across multiple vascular beds by assessing them concurrently within the same patient cohort.

## 1. Introduction

Globally, atherosclerosis remains the leading contributor to morbidity and mortality, driven mainly by complications including myocardial infarction (MI), stroke, and peripheral arterial disease (PAD) [1]. Inflammation plays a central role in atherosclerosis genesis and progression, a process that seems to be common to all vascular territories involving medium- and large-sized arteries [2].

The process of atherosclerosis initially involves the existence of endothelial dysfunction, an increased permeability to low-density lipoprotein particles, and the subsequent recruitment of circulating monocytes that transform into foam cells [3]. Local cytokine secretion is responsible for plaque formation and progression by promoting smooth muscle cell proliferation and collagen deposition, as well as cellular apoptosis and necrotic core expansion. By influencing the activity of matrix metalloproteinases, cytokines can also induce fibrous cap thinning, ultimately leading to plaque destabilization and the development of acute coronary syndromes (ACS) [3].

Galectin-3 (Gal-3) is a pro-inflammatory cytokine, a member of the lectin family of β-galactoside-binding proteins that is mainly but not exclusively secreted by macrophages [4]. Current data indicate that Gal-3 is involved in several biological processes, including cell proliferation, apoptosis, inflammation, angiogenesis, fibrosis, and host defense, thereby linking this cytokine to various diseases such as cardiovascular and renal disorders, cancer, and infections [5].

Gal-3 is involved in cardiac fibrosis and remodeling in patients with heart failure [6]. In patients with coronary artery disease (CAD), Gal-3 levels are significantly higher compared to levels in healthy individuals, and they are further elevated in those with ACS compared to chronic coronary syndromes (CCS) [7]. Furthermore, Gal-3 levels seem to correlate with the severity of CAD [7,8], although some data suggest no association with the number of affected vessels [9]. Conversely, Gal-3 correlation with atherosclerotic involvement in other vascular beds is less well documented. In patients with carotid stenosis, Gal-3 expression was lower in unstable plaques [10], while other studies show that Gal-3 correlates positively with carotid intima media thickness (IMT) values [8]. A significant influence of classical cardiovascular risk factors on IMT values has also been reported, particularly in the femoral arteries, underscoring the multifactorial nature of vascular remodeling [11]. Ursli et al. [12] found higher Gal-3 levels in patients with stable PAD as determined through clinical evaluation. There is currently no study investigating the role of Gal-3 in radial artery calcification.

Prevention remains a cornerstone of cardiovascular disease management, and so, the early identification and prediction of disease severity and extent are essential. Identifying a single biomarker capable of meeting all diagnostic and prognostic criteria would be ideal. Despite growing evidence, significant knowledge gaps remain, particularly regarding the relationship between Gal-3 and the extent of atherosclerotic disease across multiple vascular territories, assessed through systematic screening of atherosclerotic involvement in different sites within the same patient.

The aim of this study was to evaluate circulating Gal-3 levels in patients with CAD and to investigate possible correlations with coronary atherosclerotic disease severity and atherosclerotic involvement in non-coronary territories such as carotid, femoral, and radial. The role of Gal-3 in predicting short-term outcomes in ACS patients was also investigated.

## 2. Materials and Methods

### 2.1. Study Population

From January 2025 and March 2025, we prospectively enrolled consecutive patients who underwent diagnostic coronary angiography (CA) due to suspected coronary artery disease in a single center—Sibiu County Clinical Emergency Hospital. Following CA, patients were divided into three groups: the control group, the CCS group, and the ACS group, respectively. The mandatory study eligibility criteria were ≥18 years of age and a willingness to provide informed written consent. The following exclusion criteria were applied: (1) active infection; (2) chronic inflammatory or autoimmune disease; (3) active neoplasia; and (4) a decision for conservative treatment without the need for CA. In total, 75 subjects were initially included, of whom 16 were controls. The CAD population consisted of 59 subjects, of whom 1 was excluded for severe infection and 2 for incomplete data. In the CCS group, 25 patients received invasive treatment, and 7 were managed conservatively, whereas all 24 patients in the ACS group underwent invasive treatment.

The control group consisted of subjects with normal coronary arteries and without plaque in the major epicardial vessels. Gal-3 levels were compared between the controls and patients with CAD and then between patients with ACS and CCS. Subsequently, a subgroup analysis was performed between patients with high versus low Gal-3 values using the median Gal-3 value as the cut-off. CAD severity and extent were assessed for the entire population. Clinical data including demographics, cardiovascular risk factors, and history of cardiovascular disease, as well as previous medication, were collected from medical charts and interviews. Before discharge, patients underwent a multi-site vascular ultrasound scan, including carotid, femoral, and radial. Relevant echocardiography data included the left ventricular ejection fraction, the presence of diastolic dysfunction, and the left ventricular diameter.

The diagnosis of CCS was made according to the 2024 European Society of Cardiology Guidelines [13]. ACS were defined according to the 2023 European Society of Cardiology Guidelines [14] and included ST-segment elevation MI (STEMI) and non-ST-segment elevation ACS (NSTE-ACS). STEMI was characterized by continuous and persistent chest pain accompanied by an ST-segment elevation of at least 0.1 mV in two or more contiguous leads, or the presence of a new left bundle branch block on the 12-lead electrocardiogram, along with elevated cardiac biomarkers. NSTE-ACS included non-ST-segment elevation MI and unstable angina pectoris. The former was defined as the presence of ischemic symptoms with elevated cardiac enzymes without persistent ST-segment elevation, while the latter was characterized by resting angina without ST-segment elevation or positive cardiac biomarkers.

Venous blood samples were collected for a routine blood chemistry panel, including serum creatinine, complete blood count, or lipid levels. In addition, more specific laboratory data comprised cardiac biomarkers (creatin kinase-MB, cardiac troponin I, high-sensitivity cardiac troponin I, and B-type natriuretic peptide) and inflammatory biomarkers (C-reactive protein—CRP and Gal-3). For Gal-3 measurements, the samples were collected from a peripheral vein within the first 24 h after patient admission, in the Cath lab, before CA.

The study protocol was approved by the institutional review board (Sibiu County Clinical Emergency Hospital Ethics Committee no. 29469 from 2024), and it complies with the Declaration of Helsinki on human research.

### 2.2. Coronary Angiography Analysis

CAD was defined as >50% luminal stenosis in any relevant epicardial vessel with a diameter of at least 2 mm. Coronary stenosis severity was evaluated both visually and through quantitative coronary analysis methods, with the most severe degree of stenosis being taken into consideration. A threshold of 70% was used to define a significant coronary lesion, with the exception of left main or proximal left anterior descending artery, in which case the threshold was 50%. To reflect disease extent, the number of diseased vessels, and left main involvement, as well as total plaque length, were determined in >2 mm vessels with >50% stenosis. The presence of coronary calcification at any level was documented. When present, coronary calcification was categorized as mild when it was identified only after contrast injection or moderate/severe when detected before contrast injection during or irrespective of the cardiac motion [15]. Plaque length was defined as the span between the proximal and distal ends of the lesion.

The culprit lesion was identified based on electrocardiographic changes, angiographic appearance, and, in cases of uncertainty, echocardiographic wall motion abnormalities. When detected, the culprit lesion was assessed in terms of reference vessel diameter (RVD), percent diameter stenosis (DS), lesion length, intra-vessel location, bifurcation or ostial involvement, and thrombus presence. RVD was calculated as the mean between the proximal and distal references, i.e., the sites with the largest lumen diameter within 10 mm proximal and distal to the plaque. DS was determined using the following formula: [(average RVD − minimal lumen diameter)/average RVD] × 100 [16], where the minimal lumen diameter was defined as the smallest diameter within the lesion segment.

CA was performed according to the standard Judkins imaging protocol [17] using the proprietary on-site angiography machine, the Azurion 5 M20 (Philips Healthcare, Amsterdam, The Netherlands). Three experienced interventional cardiologists interpreted all CA data.

### 2.3. Galectin-3 Measurement

The blood sample was collected from a peripheral vein in a serum separating tube containing gel and a coagulation activator, allowed to stand for 30 min, and then centrifuged for 15 min at 1000× *g*. The resulting serum was then stored at −20 °C for future analysis. After all serum samples were collected, the study was performed via an enzyme-linked immunosorbent assay (ELISA) (Rayto RT3100 microplate washer and Rayto RT2100 microplate reader—Rayto Life and Analytical Sciences, Shenzhen, China), with a commercially available Human Galectin-3 ELISA kit (Thermo Fisher LSG, Waltham, MA, USA). The measurements were performed strictly according to the manufacturer’s instructions. Briefly, the method relies on the generation of antigen–antibody complexes (sandwich technique) that are subsequently detected through an enzymatic reaction. The concentration of Gal-3 correlates positively with the intensity of the colorimetric reaction measured at 450 nm. Measurements were performed in duplicate, and the results were averaged. The standard curve ranged between 0.47 and 30.0 ng/mL. The limit of detection was 0.29 ng/mL, and the intra-assay reproducibility coefficient of variation was 7.82%.

### 2.4. Vascular Ultrasound Assessment

Vascular ultrasound examination was conducted using the Philips EPIQ 7 (Philips Healthcare, Amsterdam, The Netherlands) ultrasound device equipped with a L12-5 linear-array high-resolution transducer.

IMT was measured as the distance between lumen–intima and media–adventitia interfaces. Plaque was defined according to the Mannheim consensus as a focal structure extending into the arterial lumen by at least 50% of the surrounding IMT, or measuring ≥ 1.5 mm in thickness from the media–adventitia interface to the lumen–intima boundary [18]. Plaque thickness (PT) was measured as the radial distance between the media–adventitia interface and the center of the vessel [19]. For the carotid arteries, axial sweep and longitudinal still images were acquired for the distal common carotid (far wall IMT measurement), the proximal internal carotid and bulb area (PT measurement). For the femoral arteries, axial sweep and longitudinal still images were acquired at the distal 3 cm of the common femoral (far wall IMT measurement), the distal common femoral, the femoral bifurcation, and the proximal 3 cm of the superficial and profunda femoral arteries (PT measurement). For the radial artery, longitudinal video clips and still images were obtained at the anatomical snuffbox and along a 5 cm segment proximal to this site.

**Figure 1:**
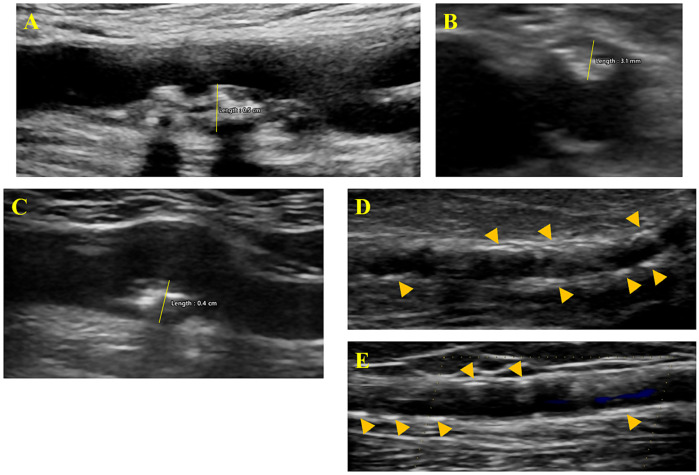
Vascular ultrasound assessment—measurement of femoral plaque thickness in longitudinal (**A**) and axial (**B**) view; measurement of carotid plaque thickness in longitudinal view (**C**); identification of distinct radial artery calcifications—arrow heads (**D**,**E**).

All ultrasound visual material was stored and examined offline in the core ultrasound laboratory by an experienced imaging technician who was not blinded to patient group allocation. For each measurement, the operator conducted three repeated acquisitions and recorded the value considered most accurate, typically reflecting an internal average. Repeated measurements generally showed a variation of less than 10%. IMT was assessed at the distal far wall of both the right and left common femoral and carotid arteries, and the average value was documented. PT was measured in the axial or longitudinal view in the femoral territory and only in the longitudinal view in the carotid territory (Figure 1). The maximal PT observed on either side (for both carotid and femoral arteries) was recorded. The radial artery was scanned for any signs of calcification, but only clearly defined hyperechoic areas with acoustical shadowing were considered positive findings (Figure 1).

### 2.5. Clinical Outcomes

Patients were followed up with at hospital visits at 1 and 3 months. When a clinical event was identified, detailed data were collected at the study site and entered into the database. Clinical events included unstable angina pectoris, MI [13], target lesion revascularization, target vessel revascularization, heart failure hospitalization, and non-culprit vessel percutaneous coronary intervention (PCI). Target lesion revascularization is defined as a repeat PCI of the target lesion, while target vessel revascularization is defined as any repeat PCI of the target vessel, including the target lesion [20].

### 2.6. Statistics

Continuous variables were tested for normality using the Shapiro–Wilk test. Variables that followed a normal distribution within their compared categories were analyzed using Student’s *t*-test and expressed as means ± standard deviations. In contrast, variables that did not meet the assumption of normality were compared using the Mann–Whitney U test and were expressed as median (interquartile range). Correlations between continuous variables were assessed using Pearson’s correlation coefficient when both variables were normally distributed, and Spearman’s rank correlation coefficient (R) when at least one variable was not normally distributed. Categorical variables were compared using the Chi-square test or Fisher’s exact test, as appropriate. We employed a multivariate analysis using binary logistic regression to identify variables independently associated with Gal-3 levels above the median. Variables that showed significant correlations with Gal-3 in bivariate analysis (either as a continuous measure or when dichotomized around the median) were included in the initial model. An exhaustive stepwise process of variable addition and removal was used to identify the optimal regression model. Continuous variables were mean-centered to reduce multicollinearity, and categorical variables were recoded into dummy variables. To estimate the 95% CI for the regression coefficients, bootstrapping with 1000 samples was conducted using the percentile method. Only variables that remained statistically significant were retained in the final model. A *p*-value of less than 0.05 was considered statistically significant.

## 3. Results

### 3.1. Baseline Characteristics

**Figure 2:**
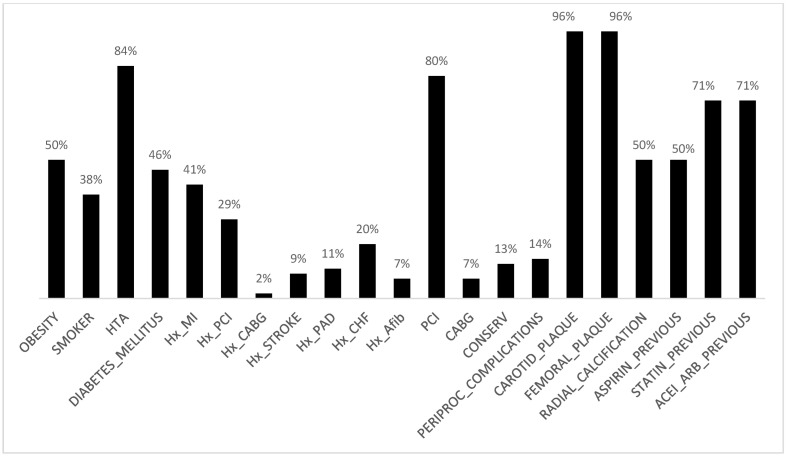
Overview of baseline characteristics in the total CAD population.

A total of 56 patients were included in the final analysis. The mean patient age was 65.9 years, while the majority were male (87.5%). Among these, ACS was documented in 24 patients (42.8%). Information on cardiovascular risk factors, comorbidities, and initial treatment administered before Gal-3 sampling is presented in Figure 2. Gal-3 concentration was significantly higher in the CAD and ACS group compared to the control group (mean: 20.72 vs. 10.06 ng/mL, *p* < 0.00001; 22.94 vs. 10.06 ng/mL, *p* < 0.00001).

Gal-3 levels were significantly lower in obese patients compared to non-obese patients (median: 17.68 ng/mL; IQR: 6.11 vs. 19.76 ng/mL; IQR: 8.26; *p* = 0.039). Gal-3 levels also showed a weak positive correlation with age (Spearman R = 0.288, *p* = 0.031).

### 3.2. Subgroup Analysis by Clinical Presentation

Concerning biochemical analyses, patients with ACS had significantly higher median Gal-3 levels (22.18 ng/mL, IQR: 7.08) compared to those with CCS (17.93 ng/mL, IQR: 5.47; *p* = 0.019), along with elevated median CRP (7.1 ng/L, IQR: 19.18 vs. 2.55 ng/L, IQR: 5.18; *p* = 0.014). No significant differences were observed between the groups in lipid profiles, hematologic parameters, renal function, cardiac ultrasound findings, CA data or vascular ultrasound measurements.

**Figure 3:**
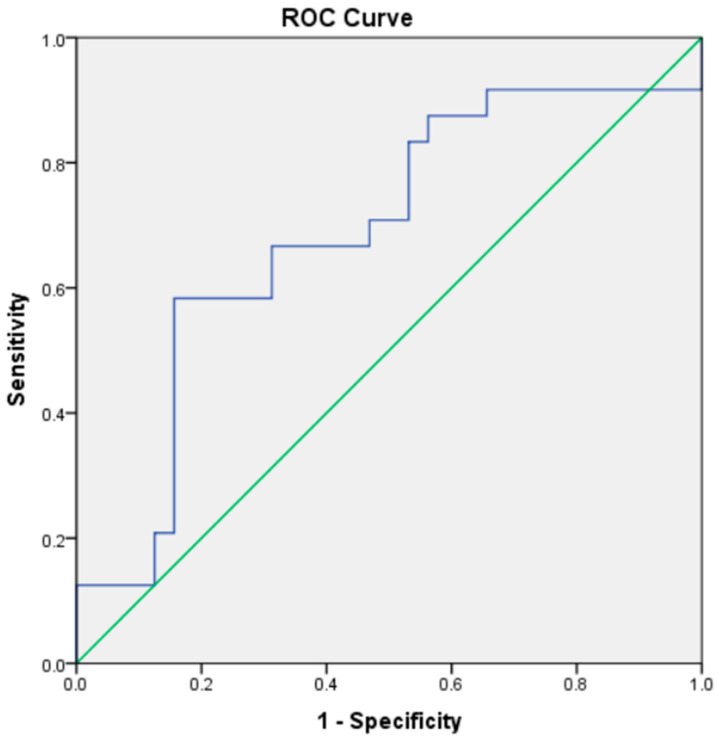
ROC plot of serum levels of Gal-3 for predicting occurrence of ACS.

We performed receiver operator characteristic (ROC) analysis to evaluate the ability of Gal-3 to distinguish ACS from CCS. The area under the curve was 0.685 (95% CI: 0.539–0.831; *p* = 0.019). We determined the optimal threshold for Gal-3 using the Youden Index. The best cut-off value was 20.18 ng/mL, corresponding to a sensitivity of 58.3% and a specificity of 84.4% (Figure 3).

### 3.3. Correlation Analysis

Gal-3 levels showed a moderate correlation with DS (R = 0.303, *p* = 0.023) and a strong positive correlation with the maximum severity of additional vessels requiring treatment (R = 0.619, *p* = 0.006). There was a signal towards higher Gal-3 values when moderate/ severe calcification was evident on CA (*p* = 0.068). Additionally, Gal-3 demonstrated a positive correlation with carotid PT (R = 0.322; *p* = 0.016).

### 3.4. Subgroup Analysis by Median Gal-3 Value

We further divided the CAD group based on the median Gal-3 value (19.07 ng/mL) (Table 1). There was increased patient age in the high Gal-3 group (68.8 years, SD = 8.09 vs. 63.07 years, SD = 11.05; *p* = 0.03). Compared to patients with Gal-3 below the median, those with Gal-3 above the median had a significantly higher DS of the culprit lesion (85.7%, IQR = 6.67 vs. 75.5%, IQR = 27.45; *p* = 0.003) and maximum severity among additional treated lesions (87.2%, SD = 5.15% vs. 72.4%, SD = 14.33%; *p* = 0.006). Numerically, more cases of moderate/severe calcification on CA were associated with a high Gal-3 value (*p* = 0.057). There was a trend toward a higher frequency of culprit lesions located in proximal vessels among patients with elevated Gal-3 levels (*p* = 0.061). Carotid PT was higher in the high GAL-3 group (31.5 mm, IQR = 11) than in the low Gal-3 group (26.15 mm, IQR = 10.25; *p* = 0.006). Similarly, femoral PT was greater in patients with Gal-3 above the median (32.4 mm, IQR = 14.65 vs. 26.45 mm, IQR = 21.40; *p* = 0.046). No significant differences were observed in other parameters reflecting CAD severity and extent, nor in carotid and femoral IMT, or radial artery involvement.

Two clinical events were recorded at follow-up, both consisting of unstable angina and occurring in the ACS and high Gal-3 groups.

### 3.5. Multivariate Analysis

The initial logistic regression model included the following variables: age, obesity, ACS presentation, culprit lesion DS, carotid PT, and femoral PT. These variables were selected based on prior bivariate analyses, in which they either showed statistically significant associations with Gal-3 (continuous or dichotomized around the median) or demonstrated trends suggestive of a possible association. The variable representing maximum severity among additional treated lesions was not included in the model, as only a limited number of patients (n = 18) presented such lesions, and its inclusion would have significantly reduced the number of analyzable cases due to the listwise deletion method employed. Hence, only variables with complete entries for all cases were considered for the analysis.

After stepwise addition and removal of variables, the optimal logistic regression model included age, DS, and ACS presentation as independent predictors of Gal-3 levels above the median. The corresponding regression coefficients, *p*-values, and percentile bootstrap-derived 95% CI are presented in Table 2.

## 4. Discussion

The involvement of inflammation in atherosclerosis is well established, with multiple pathways contributing to disease progression. Early identification and prediction of disease severity and extent through the detection of circulating inflammatory cytokines remains an area of significant clinical interest. To the best of our knowledge, this is the first study to integrate Gal-3 in the context of systemic atherosclerosis. In the present study, Gal-3, an inflammatory marker (1) was significantly higher in non-CAD compared to CAD patients, and in ACS compared to CCS patients with a good discriminatory capacity, (2) correlated positively with coronary stenosis severity, and (3) was associated with increased carotid and femoral plaque thickness.

Our findings support previous reports confirming higher Gal-3 levels in CAD patients compared to controls, as well as in ACS compared to CCS patients [7,8]. ROC and multivariate analysis confirmed the utility of Gal-3 as a predictor for ACS. Age showed a weak correlation with Gal-3 in the present study, but was an independent predictor of elevated Gal-3 levels. This finding emerges in the context of discordant data reported in the literature. Li et al. and Falcone et al. [7,9] found no correlation between Gal-3 and patient age, while data from chronic heart failure patients shows a positive correlation [21]. Nonetheless, age is recognized as a predictor of poor prognosis in CAD, and age-related increases in fibrosis and inflammation may account for our observation. Gal-3 levels were significantly lower in obese patients compared to non-obese patients in our study. In contrast to our results, Florido et al. [22] have found a strong association between body mass index and elevated Gal-3 in a cohort of over 9600 participants. This difference may, in part, be explained by the more frequent use of anti-inflammatory or modulatory therapies among our obese patients—specifically statins (82 vs. 60%) and renin–angiotensin–aldosterone system inhibitors (89 vs. 56%)—which may attenuate Gal-3 expression. Interestingly, we did not find a correlation between Gal-3 and CRP levels in our CAD population. Evidence shows that Gal-3 is mainly produced locally via activated macrophages [4], and CRP is a systemic acute-phase reactant produced via the liver in response to interleukine-6 [23]. Based on this and previous studies—some reporting only a weak correlation [7,9] and others supporting our finding [24]—we can deduce that, although both markers are linked to inflammation, Gal-3 and CRP may reflect different phases of the inflammatory response and distinct pathways involved in the pathophysiology of atherosclerosis. In our study, patients with elevated Gal-3 levels more frequently had culprit lesions located in the proximal segments of the coronary tree. Given that pathology data from Virmani et al. [25] show that plaque rupture and erosion, the two most common mechanisms underlying ACS, occur predominantly in proximal coronary segments, we hypothesize that Gal-3 may be one of the contributing factors to this pattern.

In the present study, several notable clinical distinctions emerged between ACS and CCS patients, consistent with the existing literature. In ACS patients, de novo lesions and thrombus were more frequently observed, and PCI was performed more often and more extensively, likely reflecting the need for complete revascularization in the acute setting. Moreover, ACS patients had longer hospital stays and a higher incidence of periprocedural complications, suggesting greater disease complexity and overall vulnerability. Both adverse events during follow-up occurred in patients with elevated Gal-3 levels, indicating a possible prognostic value of Gal-3 in identifying ACS patients at higher short-term risk. Interestingly, a conservative strategy was more frequently applied in our female cohort. As previous studies show that women remain at elevated risk of adverse events after PCI despite a lower disease burden and complexity, further research may be needed to provide tailored therapeutic approaches in this population, which should be explored in larger, adequately powered cohorts [26].

In the CAD group, we have found a significant correlation between Gal-3 levels and the culprit lesion and additional lesions’ maximum DS. On multivariate analysis, culprit DS was an independent predictor of elevated Gal-3. However, no significant correlation was found between Gal-3 levels and other CA parameters such as total plaque length, morphological complexity, left main involvement, and number of diseased vessels. This may suggest that Gal-3 may serve as a marker of lesion severity and not necessarily of disease extent within the coronary system. Another possible explanation for our finding is that the interaction between extra-coronary vascular disease and Gal-3 may be stronger than its association with CAD, potentially masking the influence of serum Gal-3 on CAD specifically. Both Synergy Between PCI With Taxus and Cardiac Surgery (SYNTAX) and Gensini scores were developed as CA tools to primarily assess CAD burden. The SYNTAX score evaluates anatomical complexity by accounting for lesion characteristics such as bifurcation, chronic total occlusion, calcification, and thrombus, and is primarily used to guide revascularization strategies in left main or multivessel disease. In contrast, the Gensini score provides an estimate of the extent of atherosclerotic disease, assigning weights based on both the degree of stenosis and lesion location. Although these scores offer valuable information, our study employed direct lesion-specific markers that capture both severity and complexity in a clinically driven manner. Previous work by Li et al. [7], including a mixed population of ACS and CCS patients, found Gal-3 to independently predict a high SYNTAX score, after adjustment for confounders. Similarly, Bosnjak et al. [27] reported significantly higher Gal-3 levels in patients with high SYNTAX scores compared to those with low-to-intermediate scores. However, their study was limited to patients with stable CAD and, therefore, may not fully represent a real-world clinical population. Other authors [28] demonstrated a significant correlation between Gal-3 levels and Gensini score, but after the multivariate analysis was performed, the results were not consistent. In addition, Goenka et al. [29] included 162 CAD patients and found no relation between Gensini score and Gal-3 concentration. Some studies found a positive correlation between Gal-3 and the number of diseased vessels [8,28], while Falcone et al. [9] found no differences between patients with one or two-vessel disease and those with three-vessel disease with respect to Gal-3 levels.

In the present study, we have found a positive and moderate correlation between Gal-3 concentration and carotid PT. Similar to our results, other authors found a positive association between Gal-3 and other measures of carotid subclinical atherosclerosis [30]. In the study by Lisowska et al. [8], including 333 patients with both ACS and CCS, a significant correlation was found between Gal-3 and carotid IMT values. In a large-scale cross-sectional analysis including over 5000 participants from the ARIC study, patients were divided based on Gal-3 quintile values [31]. Compared to patients in the first quintile, those in the highest quintile had higher IMT values and greater odds of carotid plaque. Conversely, conflicting results have been shown by studies investigating the role of Gal-3 in carotid plaque instability. Kadoglou et al. [10] scanned carotid plaques from 78 symptomatic or asymptomatic patients, after which carotid endarterectomy specimens were histologically analyzed. Symptomatic patients exhibited lower plaque echogenicity and, surprisingly, lower intraplaque Gal-3 concentration compared to the asymptomatic group. Interestingly, serum levels of Gal-3 did not correlate with its respective intraplaque concentration. Another study demonstrated the dual role of Gal-3 in carotid plaques—it was not only expressed by inflammatory cells in unstable plaques but was also found in highly calcified, more stable regions [32].

We have observed that patients with Gal-3 concentrations, above the median had significantly greater femoral PT than patients with low Gal-3 values. In a recent study [12], Gal-3 was measured in the serum of 311 patients with PAD stage I and II. Gal-3 concentrations were higher in symptomatic and in those with lower ankle–brachial indices, indicating more advanced disease. Conversely, Fort-Gallifa et al. [33] included patients across all stages of PAD and found no significant correlation between PAD severity and Gal-3 levels, which also applied to other inflammatory markers. Interestingly, Gal-3 levels did not differ based on the presence or absence of concomitant CAD. In a larger prospective study [34], the authors demonstrated that Gal-3 levels above the median were associated with increased five-year mortality. However, no significant differences were observed between patients with moderate versus severe PAD.

We did not find a correlation between Gal-3 levels and IMT values. One possible explanation may reside in our study population characteristics, as all patients had established CAD and the prevalence of carotid or femoral plaque was high, thus indicating the presence of more advanced atherosclerosis. IMT is generally considered an early marker of subclinical atherosclerosis and may, therefore, have limited applicability in this context. In contrast, plaque burden and severity are more reflective of disease progression and may correlate more closely with Gal-3.

We have found no correlation between Galectin-3 levels and the presence of radial artery calcification. However, a greater proportion of patients with elevated Gal-3 levels exhibited at least moderate coronary artery calcification on CA. In light of recent studies showing an association between radial and coronary calcification [35], our findings suggest that distinct pathophysiological mechanisms may underlie calcification in different vascular territories. Gal-3 has been shown to play a greater role in intimal calcification and specifically in the development of macrocalcifications [36], which may be visible during CA and are generally regarded as markers of plaque stability. However, macrocalcifications can also indicate vulnerability, especially in the form of superficial calcified plates or calcified nodules [37,38]. Intimal calcification is more characteristic of atherosclerotic processes and more prevalent in the coronary system. In contrast, the radial artery, being a medium-sized muscular peripheral artery, is more prone to medial calcification [39], a process in which Gal-3 likely plays a lesser role.

This study presents several noteworthy strengths. First, it provides a comprehensive evaluation of Gal-3 in the context of systemic atherosclerosis, integrating data from coronary, carotid, femoral, and radial vascular territories. Second, the study combines circulating biomarker analysis with detailed CA and high-resolution vascular ultrasound, enabling a nuanced assessment of the association between Gal-3 and both lesion severity and extent. Third, the inclusion of a well-characterized patient population (ACS, CCS, and angiographically normal controls) enhances the internal validity of our findings. Additionally, the real-world setting and use of median-based stratification of Gal-3 support a clinically relevant interpretation of the results. Lastly, the study explores the previously uninvestigated relationship between Gal-3 and radial artery calcification.

However, several limitations of the current study need to be addressed. First, this was a single-center study with a small sample size. Second, because of the observational, cross-sectional design, it was unable to determine a causal relation between Gal-3 and the extent of atherosclerosis. Third, although excluding known conditions associated with elevated levels of Gal-3, other potential confounders were not fully controlled (i.e., medication, heart failure, renal function). Additionally, due to the inclusion of consecutive patients, the sample was predominantly male, introducing a possible sex-related selection bias. Fourth, vascular assessment was somewhat heterogeneous (the quantification of carotid and femoral plaque; the dichotomization of radial calcification), which may have limited the strength of correlation analyses. Finally, while short-term events were recorded, a limited follow-up duration may not have captured the full clinical implications of elevated Gal-3 levels over time.

## 5. Conclusions

Gal-3 was significantly elevated in patients with ACS and correlated with both coronary lesion severity and carotid and femoral PT. Consequently, Gal-3 may constitute a valuable marker of active inflammation and systemic atherosclerotic involvement. Gal-3 may be useful in risk stratification and monitoring of atherosclerotic disease progression, though further studies are needed to confirm its clinical and prognostic utility. Our study provides integrated insights on the impact of Gal-3 across multiple vascular beds by assessing them concurrently within the same patient cohort.

## Figures and Tables

**Table 1:** Baseline, angiographic, and ultrasound features in patients with Gal-3 levels above versus below the median.

	Galectin-3 < 19.07 ng/mL	Galectin-3 > 19.07 ng/mL	*p*-Value
Age, yrs	63.07 ± 11.04	68.86 ± 8.09	0.03
Male sex, n	24 (85.7)	25 (89.3)	1
**Risk factors, n**			
Obesity	17 (60.7)	11 (39.3)	0.109
Smoking status	11 (39.3)	10 (35.7)	0.783
Hypertension	23 (82.1)	24 (85.7)	1
Diabetes mellitus	10 (35.7)	16 (57.1)	0.108
**Clinical history, n**			
Hx of MI	11 (39.3)	12 (42.9)	0.786
Hx of PCI	6 (21.4)	10 (35.7)	0.237
**Laboratory data**			
LDL-C, mg/dL	75.95 (61.25)	83.7 (62.65)	0.863
HDL-C, mg/dL	34.89 ± 9.63	34.89 ± 8.84	1
Triglycerides, mg/dL	134 (74)	150 (87.5)	0.561
Creatinine, mg/dL	0.9 (0.3)	0.9 (0.42)	0.755
Peak hs-cTnI, ng/L	4454 (5221)	3499 (5471)	0.482
BNP, pg/mL	102 (184)	159 (151)	0.39
Haemoglobin, g/dL	14.54 ± 1.35	14.2 ± 1.67	0.414
Leucocytes, 10^9^/L	8.38 ± 2.36	9.17 ± 2.51	0.229
Ne-Ly ratio	2.36 (1.45)	2.85 (2.52)	0.184
CRP, mg/dL	2.6 (9.1)	6.3 (17.1)	0.096
**Coronary angiography data**			
No. diseased vessels	2.5 (2)	3 (3)	0.249
LM involvement, n	3 (10.7)	6 (21.4)	0.469
Total lesion length, mm	67.46 ± 42.29	84.5 ± 48.35	0.166
*Calcification severity*, n			
Mild	7 (25)	4 (14.3)	0.313
Moderate/severe	13 (46.4)	20 (71.4)	0.057
Bifurcation, n	12 (42.9)	11 (39.3)	0.786
Ostial involvement, n	3 (10.7)	5 (17.9)	0.705
*Intravessel location*, n			
Proximal	11 (39.3)	18 (64.3)	0.061
Medial	14 (50)	7 (25)	0.053
Distal	3 (10.7)	3 (10.7)	1
Culprit lesion length, mm	24 (22.75)	22 (11.5)	0.7
DS, %	75.5 (27.45)	85.71 (6.67)	0.003
Thrombus presence, n	11 (39.3)	9 (32.1)	0.577
Additional treated vessels, n	7 (25)	11 (39.3)	0.252
Max. DS additional treated vessels, %	72.42 ± 14.33	87.27 ± 5.15	0.006
Total additional length treated, mm	31.71 ± 24.93	40.09 ± 34.07	0.659
Periprocedural complications, n	3 (10.7)	5 (17.9)	0.705
**Vascular ultrasound data**			
Carotid plaque, n	26 (92.9)	28 (100)	0.491
c-IMT, mm	9.78 ± 1.07	10.05 ± 1.23	0.4
c-PT, mm	26.15 (10.25)	31.5 (11)	0.006
Femoral plaque, n	26 (92.9)	28 (100)	0.491
f-IMT, mm	9.41 ± 1.29	9.16 ± 1.2	0.447
f-PT, mm	26.45 (21.4)	32.4 (14.65)	0.046
RA calcification	12 (42.9)	16 (57.1)	0.285
Days of hospitalization, n	3 (3)	4 (4)	0.577

Data are presented as n (%), mean ± SD, or median (interquartile range). BNP: brain natriuretic peptide. c: carotid. CRP: C-reactive protein. DS: diameter stenosis. f: femoral. HDL-C: high-density lipoprotein cholesterol. hs-cTnI: high-sensitivity troponin I. Hx: history. IMT: intima media thickness. LA: left atrium. LM: left main. LV: left ventricle. LVEF: LV ejection fraction. LDL-C: low-density lipoprotein cholesterol. MI: myocardial infarction. Ne-Ly: neutrophil-lymphocyte. PT: plaque thickness. RA: radial artery.

**Table 2:** Logistic regression model.

Variable	β	*p*	Percentile 95% CI for β	
			Lower	Higher
Age (mean-centered)	0.087	0.011	0.026	0.215
DS (mean-centered)	0.075	0.028	0.011	0.195
ACS	1.538	0.009	0.360	3.340
Constant	−0.699	0.082	−1.758	0.181

## Data Availability

The data presented in this study are available upon request from the corresponding author. The data are not publicly available because they are the property of Sibiu County Emergency Hospital, Sibiu, Romania.

## Abbreviations

The following abbreviations are used in this manuscript:

ACS	Acute coronary syndromes
CA	Coronary angiography
CAD	Coronary artery disease
CCS	Chronic coronary syndromes
CRP	C-reactive protein
DS	Percent diameter stenosis
ELISA	Enzyme-linked immunosorbent assay
Gal-3	Galectin-3
IMT	Intima media thickness
MI	Myocardial infarction
NSTE-ACS	Non-ST-segment elevation ACS
PAD	Peripheral arterial disease
PCI	Percutaneous coronary intervention
PT	Plaque thickness
ROC	Receiver operator characteristic
RVD	Reference vessel diameter
STEMI	ST-segment elevation MI
SYNTAX	Synergy Between PCI With Taxus and Cardiac Surgery
